# Agronomic and Physiological Traits, and Associated Quantitative Trait Loci (QTL) Affecting Yield Response in Wheat (*Triticum aestivum* L.): A Review

**DOI:** 10.3389/fpls.2019.01428

**Published:** 2019-11-05

**Authors:** Nkhathutsheleni Maureen Tshikunde, Jacob Mashilo, Hussein Shimelis, Alfred Odindo

**Affiliations:** ^1^African Centre for Crop Improvement (ACCI), University of KwaZulu-Natal, Pietermaritzburg, South Africa; ^2^School of Agricultural, Earth and Environmental Sciences, Discipline of Crop Science, University of KwaZulu-Natal, Pietermaritzburg, South Africa; ^3 ^Limpopo Department of Agriculture and Rural Development, Research Services, Towoomba Research Station, Bela-Bela, South Africa

**Keywords:** morphological traits, physiological traits, quantitative trait loci, yield gains, wheat

## Abstract

Enhanced grain yield has been achieved in bread wheat (*Triticum aestivum* L.) through development and cultivation of superior genotypes incorporating yield-related agronomic and physiological traits derived from genetically diverse and complementary genetic pool. Despite significant breeding progress, yield levels in wheat have remained relatively low and stagnant under marginal growing environments. There is a need for genetic improvement of wheat using yield-promoting morpho-physiological attributes and desired genotypes under the target production environments to meet the demand for food and feed. This review presents breeding progress in wheat for yield gains using agronomic and physiological traits. Further, the paper discusses globally available wheat genetic resources to identify and select promising genotypes possessing useful agronomic and physiological traits to enhance water, nutrient-, and radiation-use efficiency to improve grain yield potential and tolerance to abiotic stresses (i.e. elevated CO_2_, high temperature, and drought stresses). Finally, the paper highlights quantitative trait loci (QTL) linked to agronomic and physiological traits to aid breeding of high-performing wheat genotypes.

## Introduction

Wheat (*Triticum aestivum* L., 2n = 6x = 42) is the world’s third important staple food crop after maize (*Zea mays*) and rice (*Oryza sativa*). It is the most widely grown crop globally (FAOSTAT, 2018). The crop is a vital source of proteins, carbohydrates, vitamins (e.g. B1, B2, B3 and E), and mineral elements (e.g. Se, Mn, P and Cu). Wheat is used for food, industrial raw material to prepare alcoholic beverages, starch and straws, and animal feed (Nhemachena and Kirsten, 2017).

Globally, 79% of total wheat production comes from China, United States of America, Turkey, Canada, Australia, India, and Argentina (FAOSTAT, 2018) accounting to approximately 751 million tons per annum (FAOSTAT, 2018). Yield gains in wheat are currently estimated at about 0.5 to 1% per year which is below the 2.4% required to satisfy global demand (Sharma et al., 2012; Crespo-Herrera et al., 2018). In order to sustain the fast growing human population, wheat production must increase by at least 50% by 2030 (Parry et al., 2011). Additionally, the global average wheat yields must increase from 3 to 5 t ha^-1^, a growth of 1.3% yr^-1^ by 2050 to meet demands (Rosegrant and Agcaoili, 2010). Increased wheat production can be achieved through development and cultivation of genotypes with tolerance to abiotic stress and enhanced nutrient, radiation-, and water-use efficiency (WUE). Such genotypes can be developed through identification and selection of drought-adaptive and yield-influencing agronomic and physiological traits, and associated quantitative trait loci (QTL) (Chen et al., 2012; Lopes et al., 2012; Liu et al., 2015).

Grain yield response in wheat is influenced by several agronomic and physiological traits (Chen et al., 2012; Liu et al., 2015). Agronomic traits such as plant height, harvest index (HI), total biomass, number of productive tillers, grain number per spike, spike length (SL), number of kernels per spike, thousand seed weight, and grain weight per spike; and physiological traits such as canopy temperature (CT), chlorophyll content, photosynthetic rate, water-soluble carbohydrates (WSC) have contributed to grain yield improvement in wheat (Foulkes et al., 2007; Lopes et al., 2012; Beche et al., 2014; Chen and Hao 2015; Zhang et al., 2016; Gao et al., 2017). Therefore, there is a need for trait-based breeding using high performing and genetically complementary genotypes to accelerate further grain yield improvement in wheat (Reynolds & Tuberosa 2008; Chen et al., 2012; Bustos et al., 2013; Liu et al., 2015; Reynolds et al., 2017). The objective of this review is to present breeding progress in wheat for yield gains using agronomic and physiological traits. Globally available wheat genetic resources to aid identification and selection of promising genotypes possessing useful agronomic and physiological traits to enhance water, nutrient-, and radiation-use efficiency to improve grain yield potential and tolerance to abiotic stresses (i.e. elevated CO_2_, high temperature, and drought) are discussed. Finally, the paper highlights QTL linked to agronomic and physiological traits to aid breeding of high-performing wheat genotypes.

## Global Wheat Production and Yield Gains

India, Russia, China, and Kazakhstan are currently the leading wheat producers with approximately 30, 27, 24, and 12 million hectares devoted to wheat production, respectively. In terms of total production, China is the world’s leading wheat producer with approximately 131 million tons per year (FAOSTAT, 2018). Other countries such as Canada, Argentina, Ukraine, and Turkey produce a significantly large amount of wheat (FAOSTAT, 2018). Among African countries, Ethiopia Algeria, Egypt, Kenya, Morocco, and South Africa have the largest area devoted to wheat production with total production above 1 million tons per year. Variable wheat grain yield response per unit area are reported from New Zealand (9 tons/ha), Saudi Arabia (6 tons/ha), Zambia (6.6 tons/ha), Egypt (6.5 tons/ha), and China (5.4 tons/ha) in 2016 (FAOSTAT, 2018). The world average wheat yield is 2.9 tons/ha (FAOSTAT, 2018). Worldwide, about 33% countries achieved yield levels ≤ 2 tons/ha, while 21% countries recorded ≥ 3 tons/ha and 22% ≥ 5 tons/ha (FAOSTAT, 2018). Differences in yield levels is attributed to variable climatic conditions, agronomic practices, and genetic potential of cultivars.

Wheat yield gains across the major wheat producing countries are presented in **Table 1**. Genetic gains estimated through yield trials are variable among modern varieties released at various time periods compared with checks (Graybosch and Peterson, 2010; Lopes et al., 2012; Sharma et al., 2012). This variation is mainly influenced by yield-related agronomic and physiological traits (De Vita et al., 2007; Beche et al., 2014). The highest yield gains were reported in China (123 kg ha^-1^ yr^-1^), Chile (246 kg ha^-1^ yr^-1^), France (123 kg ha^-1^ yr^-1^), and Mexico (41.77 kg ha^-1^ yr^-1^), whereas relatively lower genetic progress were reported in Spain (24 kg ha^-1^ yr^-1^), Australia (25 kg ha^-1^ yr^-1^), and Siberia (15.3 kg ha^-1^ yr^-1^). Annual yield gains in Egypt, India, and Pakistan were estimated at 27.4 (0.55%), 21.4 (0.62%), 111.6 (1.13%), 32.5 (0.83%), and 18.5 kg ha^-1^ yr^-1^ (0.5%), respectively (Sharma et al., 2012). Genetic gains among CIMMYT’s spring bread wheat in the Elite Spring Wheat Yield Trial (ESWYT) in the past 15 years (i.e. 1995–2009) in 69 countries showed an annual gain of 27.8 kg ha^-1^ (0.65%) (Sharma et al., 2012). Countries such as the USA, Chile, France, and Brazil, had reportedly reached maximum limits (Brisson et al., 2010; Graybosch and Peterson, 2010; Matus et al., 2012; Beche et al., 2014).

**Table 1:** Global yield gains in wheat.

Country	Years	Yield change (tons/ha)	Mean yield increment (kg ha^-1^yr^-1^)	Genetic gain (% yr^-1^)	Reference
Canada	1885-2008	———	8.0	———	Kamran et al. (2013a)
Canada	2005-2014	2.7 -3.1	35.7	———	Perez-Lara et al. (2016)
China	1981-2008	———	51.30	0.60	Zheng et al. (2011)
China	1962-2006	———	62 kg	0.85	Xiao et al. (2012)
China	1960-2000	———	32.07 to 72.11	0.48 to 1.23	Zhou et al. (2007a)
China	1949-2000	———	13.96	0.31	Zhou et al. (2007b)
China	1940-2010	———	22.8	0.48	Sun et al. (2014)
China	1975-2007	———	103.5	1.09	Zhang et al. (2016)
China	1950-2012	5-8.5	57.5	0.7	Gao et al. (2017)
China	1945- 2010	6.08 - 7.37	66.0	———	Wu et al. (2014)
Mexico	1977-2008	———	3.5 g m^-2^ yr^-1^	0.7	Lopes et al. (2012)
Mexico	1977-2008	———	6.4 g m^-2^ yr^-1^ (HYE)	0.9	Lopes et al. (2012)
Mexico	1977-2008	———	3.0 g m^-2^ yr^-1^ (IME)	0.7	Lopes et al. (2012)
Mexico	1977-2008	———	1.0 g m^-2^ yr^-1^ (LYE)	0.5	Lopes et al. (2012)
Mexico	1994-2010	1.76 to 2.88 (LYE)	31.0	0.5	Lopes et al. (2012)
Mexico	1994-2010	3.78 to 6.02 (HYE)	———	1.0	Lopes et al. (2012)
Mexico	1966-2009	———	30 (HYE)	0.59	Aisawi et al. (2015)
Mexico	2002–2003	0.15 to 3.5	38.13 (LYE)	———	Crespo-Herrera et al. (2018)
USA	1950-2009	———	37.0	0.98	Green et al. (2012)
USA	1959-2008	———	———	1.1	Graybosch and Peterson (2010)
USA	1874-2000	———	10.4	0.48	Fufa et al. (2005)
USA	1971-2008	———	14.6	0.93	Battenfield et al. (2013)
USA	1968-2002	———	30.4	1.3	Underdahl et al. (2008)
Brazil	1940-2009	0.17 - 2.14	29.0	0.92	Beche et al. (2014)
Brazil	1999–2009	———	16.0	0.45	Beche et al. (2014)
Brazil	1998-2014	———	34.8	1.0	Bornhofen et al. (2018)
United Kingdom	1972-1995	———	0.12 Mg ha^-1^yr^-1^	———	Shearman et al. (2005)
United Kingdom	1982-2007	———	74.0	———	Mackay et al. (2011)
Spain	1988–2000	———	24.0	———	Royo et al. (2007)
Spain	1980-2009	———	24.0	0.44	Chairi et al. (2018)
Italy	1900-1990	———	19.9	———	De Vita et al. (2007)
Italy	1950-2000	———	25.6	———	Giunta et al. (2007)
Australia	1958-2007	4.1 - 6.1	25 .0	———	Sadras and Lawson (2011)
Australia	1958-2007	———	18.0	———	Sadras and Lawson (2013)
Australia	1901-2014	———	———	0.4	Flohr et al. (2018)
Australia	1958-2011	———	21.0	———	Kitonyo et al. (2017)
France	1950-1996	———	123.0	———	Brisson et al. (2010)
Siberia	1900-2000	2.18 - 3.71	15.3	0.7	Morgounov et al. (2010)
Argentina	1940-1999	———	51.0	1.17	Lo Valvo et al. (2018)
Argentina	1999–2011	———	14.0	0.18	Lo Valvo et al. (2018)
Iran	1930-2006	———	31.0	———	Joudi et al. (2014)
Iran	1930-2006	———	20.0	———	Joudi et al. (2014)
Chile	1965-2001	———	246.0	2.6	Matus et al. (2012)
France	1970–2010	0.065 - 0.137	0.114	———	Oury et al. (2012)
Turkey	1931- 2006	2.9 – 3.8	12.5	0.50	Keser et al. (2017)
Turkey	1931- 2006	0.6 – 1.8	6.1 (LYE)	0.66	Keser et al. (2017)
Turkey	1931- 2006	4.0 -5.1	18.0 (LYE)	0.49	Keser et al. (2017)
Turkey	1963- 2004	4.1 – 5.5	58.0 (HYE)	1.37	Gummadov et al. (2015)

LYE, Low-yielding environment; IME, Intermediate-yielding environment; HYE, High-yielding environment.

Genetic progress is relatively lower under low-yielding environments compared to high-yielding environments (Lopes et al., 2012; Joudi et al., 2014; Keser et al., 2017; Crespo-Herera et al., 2018). Therefore, targeted breeding for low-yielding environments (e.g. under drought stressed environments) is crucial to improve grain yield. Differences in rates of genetic progress across different breeding programmes suggest that newly developed and high-yielding genotypes possess different genetic and adaptation mechanisms to reach their yield potentials (Gummadov et al., 2015).

Genetic gains in grain yield have been attributed to development and deployment of high-yielding wheat genotypes with improved agronomic and physiological traits related with high yield potential (De Vita et al., 2007; Lopes et al., 2012; Manes et al., 2012; Aisawi et al., 2015; Zhang et al., 2016; Gao et al., 2017). For example, in Mexico genetic gains in grain yield were associated with fewer days to heading, cooler, and reduced CTs at grain filling, increased stay-green, and thousand kernel weight (Lopes et al., 2012). Similarly, significant yield increases in China resulted from increased grain number per spike, thousand kernel weight, HI, and plant height (Zhang et al., 2016). Genetic gains among CIMMYT’s spring wheat cultivars developed between 1966 and 2009 in Mexico was associated with increased above-ground dry matter and increased seed weight (Lopes et al., 2012; Aisawi et al., 2015). Further improvement in wheat genetic gains is likely to be realized through breeding for important yield-related agronomic and physiological traits.

## Use of Agronomic Traits in Phenotyping Wheat

Grain yield in wheat is influenced by several agronomic traits (Chen et al., 2012; Liu et al., 2015) which have been widely explored in wheat improvement programmes to accelerate cultivar development. Due to their high heritability and correlation with grain yield, agronomic traits can be used as indirect selection criteria during breeding and cultivar development (**Table 2**) (Chen et al., 2012; Abdolshahi et al., 2015; Liu et al., 2015; Gao et al., 2017). Moreover, it has been suggested that genetic progress in yield can be achieved if several traits conferring better agronomic and physiological performance with biotic and abiotic stress tolerance are simultaneously selected and introgressed in a single variety (Lopes et al., 2012). Some important agronomic traits that have been exploited in wheat improvement programmes to aid cultivar development and increase grain yield potential and genetic gains are discussed below.

**Table 2:** Increased (+), reduced (-), no change (#) or not yet known (?) effect of selected height reducing, photoperiod and vernalization genes on key agronomic traits in wheat.

Gene name	DH	PH	HI	BM	FLL	FLW	FLA	TKW	GNPS	GY	References
**Plant height**											
*Rht1*	?	-	?	-	?	?	?	?	+	+	Grover et al. (2018)
*Rht4*	?	-	?	-	+	+	+	-	-	-	Du et al. (2018)
*Rth4*	?	-	#	?	?	?	?	-	+	#	Liu et al. (2017)
*Rht4*	?	-	+	+	?	?	?	?	+	+	Rebetze et al. (2012a)
*Rht5*	?	+	-	?	?	?	?	-	-	-	Chen et al. (2018a)
*Rht5*	-	-	?	?	?	?	?	?	-	?	Daoura et al. (2014)
*Rht5*	+	-	?	?	?	?	?	?	-	?	Rebetze et al. (2012a)
*Rht8*	?	-	+	-	?	?	?	+	?	-	Wang et al. (2015)
*Rht8*	?	-	?	?	?	?	?	?	?	?	Rebetze et al. (2012a)
*Rht8*	?	-	?	-	?	?	?	?	?	?	
*Rht8c*	?	-	?	?	?	?	?	?	+	?	Rebetze et al. (2012b)
*Rht12*	?	-	+	+	?	?	?	?	+	+	Rebetze et al. (2012a)
*Rht13*	?	-	#	-	?	?	?	#	#	-	Wang et al. (2015)
*Rht13*	?	-	?	?	-	?	?	?	?	?	Wang et al. (2014)
*Rht13*	?	-	+	?	?	?	?	?	+	?	Rebetze et al. (2011)
*Rht13*	?	-	+	+	?	?	?	?	+	+	Rebetze et al. (2012a)
*Rht18*	?	-	+	-	?	?	?	-	+	-	Yang et al.(2015)
*RhtB1b*	?	-	?	?	?	?	?	?	+	?	Rebetze et al. (2012a)
*RhtB1b*	?	-	+	-	?	?	?	-	+	+	Liu et al. (2017)
*RhtD1b*	?	-	?	?	?	?	?	#	?	?	Wang et al. (2014)
*Rht-B1b*	?	-	?	?	?	?	?	?	+	?	Rebetze et al. (2012b)
*Rht-D1b*	?	-	+	+	?	?	?	?	?	+	Rebetze et al. (2012b)
**Vernalization**											
*Vrn-B1*	#	#	#	#	#	#	#	#	#	#	Chen et al. (2018a)
**Photoperiod**											
*Ppd-D1*	-	-	+	?	?	?	?	+	+	+	Chen et al. (2018a)
*Ppd-A1*	+	?	+	?	?	?	?	?	?	+	Royo et al. (2018)
*Ppd-A1b*	+	?	?	+	?	?	?	?	?	#	Royo et al. (2018)
*Ppd-B1b*	+	?	?	+	?	?	?	?	?	#	Royo et al. (2018)
**Combinations**											
*Ppd-A1b+ Ppd-B1b*	-	?	+	?	?	?	?	?	?	+	Royo et al. (2018)
*Ppd-D1+Rht5*	-	#	#	#	#	#	#	#	#	#	Chen et al. (2018a)
*Rth4+ RhtB1b*	?	-	+	?	?	?	?	?	+	+	Liu et al. (2017)
*Rht4+Rht8*	?	-	?	+	#	#	#	+	-	+	Du et al. (2018)
*Rht13+ Rht8*	?	?	?	?	?	?	?	?	-	?	Wang et al. (2015)
*Rht13+ RhtD1b*	?	-	?	?	?	?	?	#	?	?	Wang et al. (2014)
*Rht8c*+*Rht-B1b*	?	-	+	+	?	?	?	?	?	+	Rebetze et al. (2012b)
*Rht8c*+*Rht-D1b*	?	-	+	+	?	?	?	?	?	+	Rebetze et al. (2012b)

DH, Days to heading; PH, plant height; HI, Harvest index; BM, Biomass; FLL, Flag leaf length; FLW, Flag leaf width; TKW, Thousand kernel weight; GNPS, Grain number per spike; GY, Grain yield.

### Early Flowering and Maturity

Breeding novel wheat genotypes with early flowering and maturity is an important objective in wheat breeding programmes (Chen et al., 2016; Mondal et al., 2016; Ochagavía et al., 2018). The focus is developing early maturing wheat genotypes as an adaptive mechanism for environments experiencing terminal heat and drought stress (Motzo and Giunta, 2007; Mondal et al., 2016). Understanding the genetic factors controlling flowering time is essential to manipulate phenological development processes to improve yield potential in wheat (Royo et al., 2018). Most modern wheat genotypes incorporated vernalization and photo-period insensitive genes to promote early flowering and maturity (Chen et al., 2016). Genes conditioning vernalization namely *Vrn-A1, Vrn-B1,* and *Vrn-D1* regulate flowering and maturity in wheat (Iwaki et al., 2002). The effect of *Vrn* loci on heading and maturity, and grain yield potential are ranked as follows: *Vrn-A1* < *Vrn-B1* < *Vrn- D1* (Zheng et al., 2013; Zhang et al., 2014; Ogbonnaya et al., 2017). This resulted in increased days to heading and grain yield under optimal environments, but decreased grain yield under heat prone environments (Zhang et al., 2008; Kamran et al., 2013b; Ogbonnaya et al., 2017).

Wheat breeders have developed genotypes combining vernalization to promote early maturity and improve grain yield potential. Canadian spring wheat cultivars possessed *Vrn*-*A1* gene at a frequency of 94 % (Chen et al., 2016). In Mexico, *Vrn-D1* was identified in 66% of wheat cultivars, while *Vrn-A1*, *Vrn-B1*, and *Vrn4* were present in 41, 39, and 8% of the cultivars, respectively, either singly or in combination (van Beem et al., 2005). *Vrn-D1* allele showed the highest frequency (64%) among Chinese wheat cultivars followed by *Vrn-A1* (Zhang et al., 2008). This indicates that successful breeding using vernalization genes in wheat improvement is variable across different breeding programmes. Breeding strategies to replace the winter-type alleles, especially *Vrn*-*A1* and *Vrn*-*D1* loci associated with late heading times (Zhang et al., 2008), has been recommended to develop early-flowering cultivars for water-limited environments. Zhang et al. (2014) reported that the genotypes possessing the *Vrn-A1avrn-B1Vrn-D1a* loci would result in reduced time to anthesis and improve grain yield potential and kernel number in water-stressed environments. Contrastingly, incorporation of *Vrn-D1* is recommended in spring wheat to increase grain yield and improve adaptation to late drought and heat stress tolerance.

Photoperiod sensitive genes namely: *Ppd-D1a, Ppd-B1,* and *Ppd-A1* control photoperiod sensitivity impacting on flowering and maturation times in wheat (Gomez et al., 2014; Langer et al., 2014). The effect of selected photoperiod genes on key agronomic traits in wheat are presented in **Table 2**. Early flowering wheat genotypes with photo-period insensitivity produce high biomass and grain yield, whereas photo-period sensitivity alleles *Ppd-A1b* and *Ppd-B1b* resulted in lower yields (Royo et al., 2018). Conversely, late flowering response was induced by photo-period sensitivity due to the presence of alleles *Ppd-A1b* and *Ppd-B1b.* This produced high dry matter with little advantage in terms of grain yield potential (Royo et al., 2018). Early maturity achieved through early flowering and maturity resulted in positive genetic gains (DeVitta et al., 2007; Motzo and Giunta, 2007; Morgounov et al., 2010; Kamran et al., 2013a). In some cases, yield increase was not associated with earlier flowering in wheat (Chairi et al., 2018; Flohr et al., 2018). The limited genetic gains incorporating early maturity may be due to reduced time available for assimilate partitioning required for high grain yield development (Royo et al., 2007) partly explained by the negative association between kernel weight per spike and heading date (Zhou et al., 2007a).

The combination of *Ppd-D1* and dwarfing gene *Rht5* were reported to have negligible effect on plant growth, flowering time, spike development, and grain yield in wheat. This suggests that exploiting photoperiod-insensitive and dwarfing genes may improve grain yield by balancing flowering time and yield components (Chen et al., 2018a; Ochagavía et al., 2018). Chen et al. (2018b) reported that *Ppd-D1* and *Rht5* can shorten the duration of the reproductive phase and facilitate early flowering. *Ppd-D1* can also reduce plant height, whereas the combination of *Ppd-D1* and *Rht5* resulted in shorter plants with increased lodging resistance (**Table 2**). Furthermore, *Ppd-D1* can increase grain number from 6 to 10%, 1,000-grain weight (13 to 22%), grain yield (23 and 40%), and HI (31 and 50%) from tall and dwarf genotypes, respectively. Canadian spring wheat carrying dominant allele of *Vrn-B1*, photo-period insensitive allele of *Ppd-D1,* and height reducing allele *Rht-1* produced shorter plants and higher grain yield (Chen et al., 2016). In some breeding programmes, the photo-period sensitive gene *Ppd-D1b* is being replaced with the photo insensitive gene to develop early maturing genotypes (Kamran et al., 2013b). *Vrn-B1* can also act additively with a region on chromosome 2B near the *Ppd-B1* locus, indicating that a shorter vernalization requirement combined with the *Ppd-B1b* allele for photoperiod sensitivity may play a key role in wheat adaptation to varied environmental conditions (Addison et al., 2016). Early-maturing, high-yielding, heat-tolerant wheat genotypes with excellent adaptation to diverse environments that incorporated vernalization, photo-period, and dwarfing genes have been developed by CIMMYT and other breeding programmes globally (Chen et al., 2016; Mondal et al., 2016; Royo et al., 2018). Negative and significant correlations exist between days to flowering and grain yield potential suggesting that breeding for high yielding and early-maturing wheat genotypes can further be achieved by manipulating wheat phenology (Kamran et al., 2013b; Mondal et al., 2016). However, such genotypes should have faster growth rates and accumulate sufficient biomass production in shorter times to increase grain yield potential. Molecular markers linked to vernalization and photo-period genes useful for marker- assisted breeding have been identified in wheat (Chen et al., 2016).

### Plant Height

Breeding novel wheat genotypes with reduced plant height has increased genetic gains in wheat and significantly contributed to increased wheat productivity globally (Beche et al., 2014; Gummadov et al., 2015; Würschum et al., 2015; Zhang et al., 2016). Many wheat improvement programmes have developed wheat genotypes incorporating the dwarfing/height reducing genes namely: *Rht1 (Rht-B1b*), *Rht2 (Rht-D1b), Rht-D1c,* and *Rht8* (Zheng et al., 2011; Green et al., 2012; Lopes et al., 2012; Joudi et al., 2014; Zhang et al., 2016;Chairi et al., 2018). The genes reduce coleoptile and internode length, and plant height (Rebetzke et al., 2011; Rebetzke et al.,2012a,Rebetzke et al., 2012b) resulting in increased grain yield (Grover et al., 2018) by increasing assimilate partitioning to the ear. This resulted in higher HI and lodging resistance (Divashuk et al., 2013). Breeding progress to improve lodging resistance and grain yield in wheat resulted in plant height reduction from 130 to 60 cm in China (Gao et al., 2017), 110 to 95 cm in the UK (Berry et al., 2015), 120 to 57 cm in Italy (De Vita et al., 2007), 130 to 60 cm in Brazil (Beche et al., 2014), and from 125 to 65 cm in Spain (Royo et al., 2007) when replacing old by recent and short plant height wheat cultivars. In the USA the genetic progress of breeding for reduced plant height varied from –0.32 to –0.33% yr^–1^ and –0.37 to –0.43% yr^–1^ across varied environments (Graybosch and Peterson, 2010). Zhou et al. (2007a) and Beche et al. (2014) reported a reduction in plant height by –0.69 and –0.74% yr^–1^ among Chinese and Brazilian wheat genotypes, respectively.

To date approximately 24 height reducing genes are reported including *Rht-B1b, Rht-B1c, Rht-B1d, Rht-B1e, Rht-B1f, Rht-B1 g, Rht-D1b, Rht-D1c, Rht-D1d, Rht4, Rht5, Rht7, Rht8, Rht9, Rht12, Rht13, Rht14, Rht16, Rht18, and Rht21*. These genes regulate plant height in wheat (McIntosh et al., 2013). The effect of selected height reducing genes on selected agronomic traits are summarized in **Table 2**. However, only a few dwarfing genes have been widely utilized for improving yield in wheat (Chen et al., 2015). Knowledge regarding the function of other dwarfing genes is important for breeding (Zhang et al., 2006). Further, opportunities exist for integrating commonly used height reducing genes (i.e. Rht1, Rht2, Rht8) with other dwarfing (GAR) genes such as *Rht4, Rht5, Rht11, Rht12,* and *Rht24* to improve yield and lodging resistance (Ellis et al., 2005; Rebetzke et al., 2012a, Rebetzke et al., 2012b; Chen et al., 2018a; Mo et al., 2018). Combination of *Rht-B1e* with *Rht8 or Rht-B1b* with Rht8 reportedly improved grain yield potential (Divashuk et al., 2013). Wheat genotypes with either *Rht-B1b + Rht8c* or *Rht-D1b + Rht8c* exhibits higher grain yield, spike number, kernel number, thousand grain weight, above-ground biomass, HI, stem WSCs, chlorophyll content, and reduced plant height (Gao et al., 2017). The combination of *Rht4+Rht8* dwarfing genes has no effect on leaf length, leaf width, and flag leaf area (FLA) but resulted in reduced grain number per spike and increased 1,000–kernel weight, above-ground biomass and grain yield in wheat (Du et al., 2018). These suggested that combinations of Rht4 and Rht8 could reduce plant height to desirable levels, while improving grain yield and yield-related traits in wheat (Du et al., 2018). Similarly, combinations of dwarfing genes *Rht4* and *Rht-B1b* reduce plant height and increase grain yield due to increased grain number, greater spike number, and higher HI in wheat (Liu et al., 2017) suggesting *Rht4* can be successfully combined with *Rht-B1b* in wheat improvement to accelerate yield gains (Liu et al., 2017). Similarly, *Rht5/Rht8* improved heading date and maturity in wheat (Daoura et al., 2014) useful for breeding and cultivar development (**Table 2**). Additive gene action was reported between *Rht12* and *Ppd-D1* such that incorporation of *Ppd-D1a* enhanced flowering, and improved yield and yield-related traits of *Rht12* dwarf plants, suggesting that the combination of *Rht12* and *Ppd-D1a* may increase use of Rht12 in wheat breeding (Chen et al., 2018b). Tian et al. (2019) showed that a combination of diverse height reducing genes have already been incorporated in elite Chinese wheat genotypes. For instance, combinations of *Rht24+Rht1, Rht24+Rht2, Rht24+Rht8, Rht1+Rht8, Rht2+Rht8, Rht24+Rht1+Rht8, Rht24+Rht2+Rht8* occurred at frequencies of 86, 117, 137, 56, 77, 47, and 70%, respectively in Chinese wheat genotypes.

A dwarfing gene *Rht5* has been shown to reduce plant height by approximately 40% without affecting coleoptile length and seedling vigour (Chen et al., 2018a). However, *Rht5* can reduce SL by approximately 16.7 and 22.6%, grain number by 11.5 and 14.5%, 1,000-grain weight by 18.4 and 24.1% and grain yield by 21.5 and 35.1% and delayed ear emergence and anthesis time, thus hindering effective utilization in wheat improvement (Chen et al., 2018a). Therefore, genes promoting plant development and flowering times need to be incorporated with *Rht5* dwarf lines to exploit their potential in wheat breeding programmes. The combination of *Rht5* with other dwarfing genes to improve genetic gains in grain yield remains unexplored and un-investigated (Chen et al., 2018a). Recently, a dwarfing gene *Rht25*, with *Rht25a* representing the height-increasing allele and *Rht25b* designated the dwarfing allele were identified in wheat (Mo et al., 2018). The average dwarfing effect of *Rht25b* was found to be approximately half of the effect observed for *Rht*-*B1b* and *Rht*-*D1b*, and the effect greater in the presence of height-increasing *Rht*-*B1a* and *Rht*-*D1a* alleles than in the presence of the dwarfing alleles (Mo et al., 2018). *Rht25b* is gibberellin acid sensitive gene and shows significant pleiotropic effects on coleoptile length, heading date, SL, spikelet number, spikelet density, and grain weight (Mo et al., 2018). Therefore, *Rht25* may serve as an alternative dwarfing gene to improve wheat yield potential across diverse environments (Mo et al., 2018).

Some studies suggested that wheat plant height has reached its theoretical limit at about 70 to 80 cm, suggesting that limited progress will be achieved through further reduction in plant height (Shearman et al., 2005). As a result, plant height cannot be decreased any further to avoid risking reductions in biomass and grain yield (Berry et al., 2015). Therefore, strategic breeding that combines both plant height and grain yield to maximise yield potential and lodging resistance has been suggested (Gao et al., 2017). GAR dwarfing genes, such as *Rht4*, *Rht5*, *Rht8*, *Rht11*, *Rht12*, *Rht13*, *Rht24*, *and Rht25* have the potential to reduce plant height further (Rebetzke et al., 2012a, Rebetzke et al., 2012b; Chen et al., 2018a; Mo et al., 2018). These genes (i.e. *Rht4*, *Rht5*, *Rht8*, *Rht11*, *Rht12*, *Rht13*, *Rht24*, *and Rht25*) have negligible effects on biomass production, whereas some (i.e. *Rht4*, *Rht12*; *Rht13*; *Rht24*) can increase above-ground biomass, kernel weight, and grain yield (Rebetzke et al., 2012a; Rebetzke et al., 2012b; Yang et al., 2015; Würschum et al., 2017; Tian et al., 2019). The *Rht24b* allele is already used in combination with the two *Rht‐1b* semi‐dwarfing genes in wheat breeding (Würschum et al., 2017; Tian et al., 2019). As a result, *Rht24* utilization has increased in European countries, China and the USA, indicating that wheat breeders have actively selected for this locus for cultivar development to improve lodging resistance and grain yield potential (Würschum et al., 2017; Tian et al., 2019). *Rht24* occurs at a frequency of about 84.2% than other important dwarfing alleles in elite wheat varieties in China and usually couples with *Rht2* or *Rht8* (Tian et al., 2019). Similarly, Würschum et al. (2017) also showed that *Rht24* occurred at high frequency of approximately 67% compared with GR genes and *Rht8* in >1,000 wheat varieties originating mainly from Europe. However, while transferring height reducing genes to well-adapted wheat genotypes, attention should be directed to selection of the most suitable adapted parents as the effect of the gene vary with different genetic backgrounds (Yang et al., 2015). Additionally, very limited information is available detailing the effect of dwarfing genes on wheat physiological processes which may limit effective breeding targeting such traits.

### Harvest Index

HI has accelerated breeding for improved grain yield potential in wheat. For example, HI in wheat improved from approximately 0.25–0.44 (Gao et al., 2017) and 0.26–0.55 (Zhang et al., 2016) in China, 0.42–0.46 in the USA (Green et al., 2012), 0.26–0.42 in Spain (Royo et al., 2007), 0.21–0.43 in Australia (Flohr et al., 2018), 0.41–0.43 in Italy (Giunta et al., 2007), and 0.28–0.36 in Turkey (Gummadov et al., 2015). Additionally, gains in HI increased at an average of 0.51 and 0.63% yr^-1^ in China (Zhou et al., 2007a; Gao et al., 2017), 0.19% yr^-1^ in Italy (Giunta et al., 2007), and 0.11 and 0.002% yr^-1^ in Australia (Sadras and Lawson, 2011; Flohr et al., 2018). Despite significant improvement in HI, the trait has remained at approximately 0.55 which is below a theoretical limit of 0.62 (Gaju et al., 2009). In China, HI of some widely cultivated cultivars released between 1945 and 2010 have reportedly reached their theoretical maximum limit suggesting future gains in yield may depend on achieving greater harvest biomass production, while maintaining HI (Shearman et al., 2005). Linear and positive relationship was observed between HI with grain yield over time suggesting that HI can improve yield gains even further (Zheng et al., 2011).

### Biomass Production

Increased biomass has resulted in grain yield improvement in wheat. The increase in biomass has been largely attributed to higher photosynthetic rate, stomatal conductance, leaf chlorophyll content and improved radiation-use efficiency (Bustos et al., 2013). It has been suggested that further improvements in grain yield can be achieved by increasing photosynthetic capacity by optimizing biomass production while maintaining lodging resistance (Beche et al., 2014). Several studies showed that biomass contributed significantly to increased grain yield (Shearman et al., 2005; Xiao et al., 2012; Bustos et al., 2013; Aisawi et al., 2015; Gao et al., 2017), whereas others studies indicated very little contribution of this trait (Royo et al., 2007; Tian et al., 2011; Zheng et al., 2011; Sun et al., 2014; Zhang et al., 2016). In China, Gao et al. (2017) reported that biomass accumulation significantly increased by 0.39% yr^-1^ or 62.6 kg ha^-1^ yr^-1^, among new Chinese wheat cultivars. Reynolds et al. (2017) reported that crossing complementary genotypes exhibiting high biomass and HI may improve yield gains in wheat than crossing only high yielding genotypes. Zheng et al. (2011) also reported that further increases in above-ground biomass and HI may continue to contribute to grain yield improvement in genotypes within optimum plant height. However, the negative relationship between plant height with HI and biomass may offset such gains. In some instances, positive association has been reported (Aisawi et al., 2015) which further suggests manipulation of this trait can improve genetic gains in grain yield even further.

### Kernel Weight

Grain yield improvement has been significantly associated with increased thousand kernel weight (TKW) (Zhou et al., 2007a; Morgounov et al., 2010; Tian et al., 2011; Zheng et al., 2011; Lopes et al., 2012; Aisawi et al., 2015). On the contrary, non-significant contribution of TKW were reported (Shearman et al., 2005; Royo et al., 2007; Acreche et al., 2008; Brisson et al., 2010; Xiao et al., 2012) especially under heat stress condition limiting the selection response for this trait under low-yielding environments (Sharma et al., 2012; Lopes et al., 2012). Improvement in TKW ranged from 39 to 55 g (Gao et al., 2017) and 29 to 49 g (Zhang et al., 2016) among old landrace varieties and newly-developed Chinese wheat genotypes. Similarly, Giunta et al. (2007) reported TKW of 33 to 54–55 mg in old cultivars and 41 to 57 mg in modern wheat cultivars. In the USA, Underdahl et al. (2008) also reported improvement in TKW ranging between 20.4 to 33.6 g for old (i.e. 1973) and newly released (i.e. 2004) cultivars, respectively. Additionally, Gao et al. (2017) and Underdahl et al. (2008) reported genetic gains of 0.35% yr^-1^ (0.18 g yr^-1^) and 0.3% yr^-1^ for TKW among Chinese and American wheat genotypes, respectively. Similarly, Beche et al. (2014) reported increased TKW of 0.03 g yr^-1^ among Brazilian wheat genotypes. TKW is reportedly linear with moderate to high correlation with grain yield (Morgounov et al., 2010; Zheng et al., 2011; Qin et al., 2015; Gao et al., 2017) suggesting selection of heavier grains could be highly effective for improving wheat yield gains. As a result, increasing grain weight potential at specific positions within the spikelet has been suggested (Calderini and Reynolds, 2000), rather than breeding for higher TKW. Breeding for high grain number and TKW in the same genotype has been reported to be difficult due to trade-offs. Gaju et al. (2009) suggested trade-off can be minimized by selecting genotypes with higher number of spikelets per spike (SPS). These authors showed that genotypes with high spikelet number resulted in spikes with higher grain number and heavier TKW. An alternative approach involving crossing of suitable genotypes possessing contrasting grain number and grain weight to combine both traits in the progeny has also been proposed by Bustos et al. (2013). These authors reported an increase in grain yield combining both traits confirming the possibility that crossing genotypes expressing high grain number with those expressing high TKW (and with similar yield and biomass) might be a useful strategy to increase yield potential in wheat.

### Number of Grains Per Spike

The number of grains per spike has been identified as an important trait for improving grain yield (Yu et al., 2014; Alonso et al., 2018; Würschum et al., 2018; Liu et al., 2018c). Yield gains resulting from improvement in grain number has been reported, (Tian et al., 2011; Flohr et al., 2018; Liu et al., 2018c) suggesting successful selection in wheat breeding (Xiao et al., 2012; Aisawi et al., 2015); whereas in some instances it was not associated with genetic progress in grain yield (Zhou et al., 2007a; Xiao et al., 2012; Gao et al., 2017). Chairi et al. (2018) reported grain yield increases of 24 kg ha^-1^ y^-1^ (0.44% yr^-1^) between 1980 and 2003 attributed to high number of kernels spike^-1^ (0.24 kernels spike^-1^ yr^-1^) in Spain. Similarly, Joudi et al. (2014) reported improvement of grain number per spike of 35 grains m^-2^ yr^-1^ through breeding and selection spanning over 50 years in Iran. Grain number among Brazilian wheat genotypes was increased by 77.89 grains yr^-1^. In China grain number per spike varied between 26 for landraces developed in 1941 to 38 for improved wheat genotypes released between 2007 and 2011 (Zhang et al., 2016). Among wheat cultivars developed in the USA, number of grains per spike varied between 25 and 38 for old and modern cultivars (Green et al., 2012). Although indirect selection for genotypes with a higher grain number has been effective, the negative correlation between the number of grains per spike and thousand kernel weight suggests that further increases in number of grains would be partially offset by reductions in grain weight (Sadras and Lawson, 2011; Bustos et al., 2013). Therefore, an increase in the number of spikelets can be selected concurrently with increased SL, to offset an increase in spike compactness (SC) (Würschum et al., 2018). The relationship between grain yield and grain number is reportedly curvilinear in some instances suggesting that the strategy for increasing grain yield through higher grain number could be less efficient (Sadras and Lawson, 2011; Bustos et al., 2013). On the contrary, the linear relationship reported between grain number per spike and grain yield suggest the likelihood of this trait in improving grain yield potential in some instances (Tian et al., 2011; Qin et al., 2015).

### Spike Fertility

Spike fertility (SF) is a grain yield component that influences the increase in the number of kernels per spike (Reynolds et al., 2017; Würschum et al., 2018). For instance, selection for SF either solely or in combination results in higher grain yield, than selecting for high yield alone (Alonso et al., 2018). Increase in the number of kernels per spike were attributed to increased SF (Würschum et al., 2018). In addition, SF is a highly heritable trait, controlled by several genes with additive effects (Alonso et al., 2018). Number of kernels per spike and spikelet fertility are significantly and positively correlated but negatively correlated with kernel weight (Würschum et al., 2018). These suggested increase in either number of kernels per spike and spikelet fertility will likely reduce TKW and grain yield potential in wheat. This effect suggests that improvement in grain yield can be achieved through an integrated approach targeting several yield-component traits (Würschum et al., 2018). Novel wheat genotypes possessing large spikes (e.g. high assimilate partitioning to spike, long rachis, high spikelet number per spike, high fertile florets per spikelet) are maintained by CIMMYT useful of breeding (Gaju et al., 2009). In China, wheat genotype Zhongmai 895 released in 2012 with a yield potential at 8,906 kg ha^-1^ was derived from ‘Zhoumai 16’ x ‘Liken 4’. Zhoumai 16 was developed from ‘Yumai 21’ x ‘Zhou 8425B’ whereby Zhou 8425B is characterized by large spikes and high TKW (Gao et al., 2017), demonstrating the feasibility of incorporating large spikes in wheat improvement programmes.

Other spike characteristics useful for breeding include SL, SPS, and SC (Chairi et al., 2018; Würschum et al., 2018). The number of kernels per spike is positively and moderately correlated with SPS and SL; whereas SL is positively correlated to SC (Würschum et al., 2018). Spike characteristics are highly heritable traits with heritability values of 0.90 for SL, 0.92 for SPS, 0.93, and 0.67 for SC (Würschum et al., 2018). De Vita et al. (2007) reported that SL and SPS did not improve grain yield potential of durum wheat cultivars released in Italy between 1900 and 1990. The contribution of other spike traits as selection criterion for advancing grain yield genetic gains in wheat are yet to be explored.

### Number of Productive Tillers

Number of productive tillers defined as the number of tillers that produce spikes and seeds is a key agronomic trait that affect biomass production and grain yield potential in wheat (Tausz-Posch et al., 2015). Wheat genotypes with reduced tillering capacity are more productive than free-tillering genotypes under drought stressed conditions (Naruoka et al., 2011; Wang et al., 2016; Houshmandfar et al., 2019) due to reduced sterile spikelets (Gaju et al., 2014). Contrastingly, Sadras and Rebetzke (2013) reported that lines possessing the free-tillering allele showed increased tiller production which was related to increased grain yield potential under high-yielding environments. Several tiller inhibition genes (Duggan et al., 2005; Mitchell et al., 2012; Wang et al., 2018) and tiller promoting genes (Naruoka et al., 2011) have been identified in wheat useful for improving wheat grain yield. *Tin1* tiller inhibition gene can increase grain number per spike (Duggan et al., 2005; Gaju et al., 2014) and HI from 0.31 to 0.35 (Motzo et al., 2004). Therefore, the introgression of the *Tin1* gene into modern wheat germplasm may offer opportunities to increase grain number per spike, grain m^-2^, HI, and ultimately grain yield improvement in wheat (Gaju et al., 2014).

### Leaf Morphology and Its Component Traits

Optimal flag leaf morphology can improve light absorption, which improves photosynthesis and grain yield potential (Liu et al. 2018a). Leaf traits such as flag leaf angle (FLAN), flag leaf width (FLW), flag leaf length (FLL), the ratio of length/width of flag leaf (FLR), and FLA may be useful for improving grain yield in wheat. FLL, FLW, and FLA are correlated with some important agronomic traits (Liu et al., 2018 a, b). Additionally, FLL, FLW, and FLA have been reported to be significantly and positively correlated to SL, grain weight per spike, and grain number per spike (Fan et al., 2015; Liu et al. 2018a; Liu et al. 2018b Wu et al., 2016; Zhao et al., 2018) indicating leaf traits influence yield-related traits (Liu et al., 2018a). Wheat genotypes with relatively larger flag leaf size tends to produce more kernel number per spike (Zhao et al., 2018), suggesting appropriate flag leaf size could promote development of high grain yield potential. FLA is reportedly the most yield contributing trait, followed by FLW and FLL (Fan et al., 2015). In the USA, Balota et al. (2017) reported that yield gains in soft red winter wheat genotypes developed between 1919 to 2009 were associated with reduced leaf area suggesting yield increases were achieved through selection of smaller leaf size. Broad-sense heritability for FLAN, FLW, FLL, FLR, and FLA are reportedly higher (> 70%), indicating that flag leaf traits could be targeted for breeding and cultivar development (Wu et al., 2016; Liu et al., 2018a).

### Root and Root-Related Traits

Root system development and its component traits are useful attributes to extract water from deeper soil profile under water-limited conditions. These traits are associated with heat and drought stress adaptation (Lopes and Reynolds, 2010; Pinto and Reynolds, 2015; Merchuk-Ovnat et al., 2016). Therefore, root and root-related traits can be integrated in wheat improvement programmes for cultivar development. Enhanced root development (based on root dry weight analysis) in wheat is negatively correlated with WSCs under drought-stress condition indicating extensive root development enhances drought tolerance by accumulation of carbohydrates (Lopes and Reynold, 2010; Mkhabela et al., 2019). Root-related traits such as root: shoot ratio negative relationship with agronomic traits such as plant height, number of tillers, shoot biomass, thousand grain weight, and grain yield. This suggests that reduced root development may be negatively associated with yield gains under water-stressed environments (Bai et al., 2013; Mathew et al., 2018). Root traits including root biomass are positively correlated to number of grains per spike and grain yield (Ehdaie et al., 2016). This suggests that root development contributes to yield and yield-related traits expression. Yield gains under water-stressed conditions have been attributed to enhanced capacity of new breeds of wheat to extract water from deeper soil profile (Pask and Reynolds, 2013). The ability to extract water from deeper soil horizon during moisture deficit condition is attributed to development of deeper and vigorous root system. This is accompanied by enhanced stomatal conductance and transpiration rates, and reduced CT resulting in higher grain yield and biomass production (Pask and Reynolds, 2013). Despite the direct influence of root and root-related traits on drought tolerance there is limited information on their role on yield gains in wheat breeding programs. Further, whether changes in yield gains could be related to root component traits in wheat is not clearly understood.

## Phenotyping Based on Physiological Traits in Wheat

Knowledge of physiological traits associated with genetic gains in yield is important for breeding (Beche et al., 2014; Aisawi et al., 2015; Zhang et al., 2016). It has been reported that breeding wheat genotypes incorporating physiological traits can improve grain yield genetic gains by approximately 50% (Reynolds et al., 2012). Physiological traits that have contributed to grain yield improvement in wheat are discussed below.

### Canopy Temperature

CT has significantly played a key role in improving yield potential in wheat (Lopes and Reynolds, 2010; Lopes et al., 2012; Gao et al., 2017). Cooler plant canopy during mid-grain filling is linked to higher drought tolerance and yield under water-limited condition (Thapa et al., 2018). Breeding genotypes with reduced CT resulted in lowering this trait from 30 to 29° C in CIMMYT spring bread wheat programme spanning over 30 years (Lopes et al., 2012). Similarly, genetic gains in CT increased by 0.12% yr^-1^ among Chinese wheat cultivars (Gao et al., 2017). Further, a significant negative linear relationship existed between CT and grain yield with year of cultivar release (Lopes et al., 2012; Pinto et al., 2017; Thapa et al., 2018) indicating possibilities to reduce CT further to increase grain yield in wheat (Lopes et al., 2012).

### Chlorophyll Content

Chlorophyll content is useful trait for breeding for high grain yield potential in wheat. Several reports have shown some breeding progress incorporating this trait with new wheat cultivars showing slightly high chlorophyll content than old cultivars (Beche et al., 2014). Increased post-anthesis chlorophyll content is positively and moderately correlated with HI, leaf CT, water soluble carbohydrates, and grain yield (Lopes et al., 2012; Gao et al., 2017). The stay-green trait which is related to retention of chlorophyll content has been identified as a key target trait for improving light interception and utilization and can contribute to increased wheat yield (Cossani and Reynolds, 2012). Similarly, Lopes and Reynolds (2012) showed that stay-green was correlated with yield under heat stress and heat combined with drought in spring wheat. Therefore, selection for stay-green trait in promising wheat genotypes will likely increase the rate of genetic progress for adaptation of wheat under both well-watered and water-limited environments (Christopher et al., 2018). The linear association reported between stay-green trait and grain yield improvement suggest the latter can be targeted for cultivar development (Lopes et al., 2012; Beche et al., 2014).

### Enhanced Photosynthetic Capacity

Understanding changes in photosynthetic capacity among elite wheat genotypes is important for improving yield gains (Parry et al., 2011; Zheng et al., 2011; Reynolds et al., 2012). In China, changes in leaf photosynthesis were not associated with improved grain yield in 70 years of wheat improvement (Chen and Hao 2015). The lack of correlation between genetic changes in photosynthetic rate and yield increase suggests that leaf photosynthesis does not limit/improve grain yield development or that cultivar development has not specifically targeted improved photosynthetic capacity. As result, determinants of sink strength should be targeted for increasing yield rather than selection for higher photosynthetic rates under drought stress condition (Chen and Hao, 2015). Conversely, genetic gains in rates of post-anthesis net photosynthesis were closely and positively correlated with grain yield (Zheng et al., 2011; Beche et al., 2014). Fischer et al. (1998) also reported that wheat yield gains were associated with higher stomatal conductance and increased photosynthetic rate. Other photosynthesis-related traits such transpiration rate, stomatal conductance or WUE were reported non-influential on yield development (Chen and Hao, 2015) whereas other studies (i.e. Sayre and Rajaram, 1997; Beche et al., 2014) reported improved genetic gains. CIMMYT’s heat and drought tolerant wheat genotypes showed genetic gains in yield with correlation to physiological traits (Lopes et al., 2012). Positive relationships have been reported between photosynthetic rate and chlorophyll content suggesting increased chlorophyll content improves photosynthetic efficiency (Zhang et al., 2009). Positive correlations have been reported between photosynthetic rate with stomatal conductance and biomass production (Beche et al., 2014) suggesting enhanced stomatal conductance and photosynthetic rate increases the rate of biomass accumulation (Parry et al., 2011). To improve photosynthetic efficiency, crosses can be conducted between adapted wheat cultivars with those exhibiting high-photosynthetic rates. Higher yield levels can be achieved by integrating photosynthesis related traits (e.g. stomatal conductance and transpiration rate) with yield-related agronomic traits (Zhang et al., 2016) to develop genotypes with higher yield potential (Rebetzke et al., 2013).

### Water Soluble Carbohydrates

WSC significantly improved yield gains in wheat (Shearman et al., 2005; Foulkes et al., 2007; Gao et al., 2017). In addition, significant correlations with grain yield have been reported between radiation-use efficiency and WSC in wheat (Shearman et al., 2005; Foulkes et al., 2007), which suggested that genetic gains in wheat yield is driven by improved growth rate due to increased accumulation of WSC (Shearman et al., 2005). Sadras and Lawson (2011), and Gao et al. (2017) reported genetic gains in WSC of 0.12 and 0.81% yr^-1^ among Australian and Chinese wheat genotypes, respectively. Genotypes with high WSC are commonly shorter, flower and mature earlier, and produce significantly fewer tillers than those with low WSC (Rebetzke et al., 2008), suggesting cultivar development targeting incorporation of plant height (e.g. *Rht-B1*) and/or anthesis date genes (e.g. *Ppd1*) resulted in improvement of WSC. In addition, wheat genotypes with high WSC produce more fertile tillers, reduced days to anthesis, increased biomass, grain number, and grain yield than genotypes with low WSC (Rebetzke et al., 2008). Grain weight is high in genotypes with high WSC during early grain filling stages, indicating that more available assimilates contribute to higher grain weight potential (Dreccer et al., 2009). Further, WSC accumulation ability and remobilization efficiency in drought tolerant cultivars is much higher than those in sensitive wheat genotypes (Hou et al., 2018) suggesting increased WSC enhances drought tolerance in wheat (Hou et al., 2018). It has been suggested that cultivar development may have targeted improvement of photosynthetic efficiency which has driven increases in number of grains and a larger source for grain filling through increases in stem WSC (Shearman et al., 2005). Moderate to high heritability of WSC (Ruuska et al., 2006; Rebetzke et al., 2008) suggest breeding for either high or low concentration of WSC is possible.

## Wheat Genetic Resources for Improving Wheat Grain Yield Genetic Gains

Exploration of wheat genetic resources is useful to identify sources of variation for agronomic and physiological traits and discovery of new alleles for improving grain yield potential (Zhang et al., 2016; Reynolds et al., 2017; Liu et al., 2018c). Wheat genetic resources including landrace varieties, synthetic cultivars, and wild relatives including *Triticum tauschii* L. and wild emmer wheat (*T. dicoccoides* Korn (2n = 28, AABB) possess useful source of alleles for enhancing drought tolerance and improving yield and its component traits (Gororo et al., 2002; Moeller et al., 2014; Cossani and Reynolds, 2015; Gaju et al., 2016; Merchuk-Ovnat et al., 2016; Pinto et al., 2017; Reynolds et al., 2017; Liu et al., 2018c). In China, about 48 improved wheat genotypes released between 2011 and 2016 were developed using synthetic wheat (Liu et al., 2018c). Synthetic wheat genotypes with high biomass and yield expression, and physiological traits such as higher leaf photosynthetic rate and lower leaf transpiration rates have been identified (del Blanco et al., 2000; Reynolds et al., 2015; Pinto et al., 2017). Modern high-yielding cultivars that incorporated genes from synthetic-wheat tend to have higher gas exchange rates compared to older cultivars (De Vita et al., 2007; Sadras and Lawson, 2011; Xiao et al., 2012; Beche et al., 2014). Cossani and Reynolds (2015) identified a set of advanced wheat lines derived from synthetic hexaploid wheat with high levels of heat tolerance incorporating several drought adaptive mechanisms such as such as higher crop growth rate, increased WSCs storage in stems, cooler CT, and spectral indices which are related to pigment composition, photo-protective mechanisms, and increased radiation use efficiency. These traits result in increased number of grains, growth of taller stems with a greater WSCs storage capacity significantly related to increased kernel weight (Cossani and Reynolds, 2015). Synthetic wheat genotypes are developed by crossing wild wheat (*Aegilops tauschii*) with durum wheat (T. durum Desf.) (Cossani and Reynolds, 2015). Tetraploid wheat (*T. turgidum* L.) is also identified as a useful genetic resource for wheat breeding possessing functional genes that surpass the early maturity effect caused by the early flowering allele *Ppd-A1a* found in *T. turgidum* L. (ssp. turgidum conv. pyramidale) (Nishimura et al., 2018). Wild emmer wheat is also considered a promising source of useful genes for improving stress resistance, grain protein quality and quantity, and micronutrient concentrations in domesticated wheat (Xie and Nevo, 2008; Merchuk-Ovnat et al., 2016). Wheat genotypes with drought and heat tolerance that incorporated genes from landraces and synthetic wheat have been developed for cultivation in arid and semi-arid environments to boost grain yield potential (Lopes et al., 2012; Cossani and Reynolds, 2015; Mondal et al., 2016; Pinto et al., 2017; Crespo-Herrera et al., 2018). Further, molecular, and physiological characterization of wheat genetic resources is useful to increase the probability of achieving cumulative gene action to improve yield gains (Ortiz et al., 2008; Reynolds et al., 2017).

## Quantitative Trait Loci (Qtls) Associated With Agronomic and Physiological Traits in Wheat

QTL mapping of agronomic and physiological traits is important for marker-assisted breeding in wheat (Huang et al., 2003; Lozada et al., 2017; Li et al., 2018; Liu et al., 2018b). Agronomic and physiological traits are controlled by a number of QTL (**Tables 3**, **4**). Several multiple QTLs linked to agronomic traits have also been identified such as *QTLs QTn.ipk-5D*, *QTn.ipk-2D*, *QTn.ipk-3B*, and *QTn.ipk-1B* which are associated with productive tiller number (Huang et al., 2003). QTL *QFlt.dms-2D*, *QFlt.dms-5B*, *QFlt.dms-2D*, *QFlt.dms-7A*, and *QFlt.dms-6B.2* are linked to days to flowering; whereas, QTLs *QMat.dms-2D*, *QMat.dms-2D*, *QMat.dms-7A.2*, and *QMat.dms-4A.1* are associated with days to maturity (Perez-Lara et al., 2016). About 40 QTL’s associated with kernel morphological traits such as kernel length, kernel width, kernel thickness, kernel length/width ratio, kernel length/thickness ratio, and kernel width/thickness ratio have been recently mapped in wheat (Chen et al., 2019). New QTLs linked to FLL, FLW, and FLA were recently identified and mapped in wheat (Liu et al., 2018a, Liu et al., 2018b). QTLs for root and root-related traits have also been mapped in wheat (**Table 3**) (Hamada et al., 2012; Bai et al., 2013). QTL’s for root component traits were reported to be co-located with those controlling plant height, indicating that wheat genotypes possessing height reducing genes (i.e., Rht) may have reduced root growth under water-stressed environments (Bai et al., 2013). Further, QTLs controlling canopy temperature reportedly affects root system development (Ren et al., 2010). The identified QTLs can be transferred to new or well-adapted cultivars to improve yield in wheat (Zhang et al., 2018).

**Table 3:** Key agronomic traits and their quantitative trait loci (QTLs) in wheat.

Trait	QTL name	Chromosome location of QTL	References
Days to flowering & maturity	*QEps.dms-1B1*	1B	Kamran et al. (2013b)
	*QEps.dms-1B2*	1B	Kamran et al. (2013b)
	*QEps.dms-5B1*	5B	Kamran et al. (2013b)
	*wPt-741686*	7A	Ogbonnaya et al. (2017)
Days to flowering	*QFlt.dms-4A1*	4A	Kamran et al. (2013b)
	*QEps.dms-1B1*	1B	Kamran et al. (2013b)
	*QEps.dms-5B1*	5B	Kamran et al. (2013b)
	*QFlt.dms-4A.1*	4A	Kamran et al. (2013b)
	*VRN-A1*	5A	Ogbonnaya et al. (2017)
	*wPt-2822*	6A	Ogbonnaya et al. (2017)
	*VRN-B1*	5B	Ogbonnaya et al. (2017)
	*VRN-D1*	5D	Ogbonnaya et al. (2017)
	*wPt-741686*	7A	Ogbonnaya et al. (2017)
Plant height	*QPH.cgb-1B.1*	1B	Wu et al. (2012)
	*QPH.cgb-2D.1*	2D	Wu et al. (2012)
	*QPH.cgb-4D*	4D	Wu et al. (2012)
	*QPH.cgb-6B.6*	6B	Wu et al. (2012)
	*Rht24*	6A	Würschum et al. (2017)
	*qRht.3A*	3A	Würschum et al. (2017)
	*qRht.2D*	2D	Würschum et al. (2017)
	*Ppd‐D1*	2D	Würschum et al. (2017)
	*wPt-1038*	5A	Ogbonnaya et al. (2017)
Harvest index	*qHI-2B*	2B	Ehdaie et al. (2016)
	*qHI-2D*	2D	Ehdaie et al. (2016)
Biomass	*qPBio-7D*	7D	Ehdaie et al. (2016)
	*qPBio-2D*	2D	Ehdaie et al. (2016)
	*qPBio-3A*	3A	Ehdaie et al. (2016)
	*qPBio-6B2*	6B	Ehdaie et al. (2016)
	*qSBio-2D*	2D	Ehdaie et al. (2016)
	*qSBio-6B*	6B	Ehdaie et al. (2016)
	*qSBio-2A*	2A	Ehdaie et al. (2016)
	*qSBio-7D*	7D	Ehdaie et al. (2016)
	*qPBio-4A*	4A	Ehdaie et al. (2016)
	*qPBio-6B1*	6B	Ehdaie et al. (2016)
	*qSBio-1B*	1B	Ehdaie et al. (2016)
	*qPBio-1B*	1B	Ehdaie et al. (2016)
Thousand grain weight	*QTGW.cgb-2D.2*	2D	Wu et al. (2012)
	*QTGW.cgb-3A.4*	3A	Wu et al. (2012)
	*IWB36400*	2A	Sukumaran et al. (2018)
	*IWB2414*	2B	Sukumaran et al. (2018)
	*IWB42660*	2B	Sukumaran et al. (2018)
	*IWB9110*	1B	Sukumaran et al. (2018)
	*IWB23810*	3D	Sukumaran et al. (2018)
	*IWB8975*	5A	Sukumaran et al. (2018)
	*IWB65783*	6A	Sukumaran et al. (2018)
	*IWB52005*	6B	Sukumaran et al. (2018)
	*IWB18267*	7D	Sukumaran et al. (2018)
	*IWB15280*	7D	Sukumaran et al. (2018)
	*D3956560*	2A	Würschum et al. (2018)
	*D1296988*	3D	Würschum et al. (2018)
	*Rht*-*D1*	4D	Würschum et al. (2018)
	*Rht*-*B1*	4B	Würschum et al. (2018)
	*wPt-2315*	1B	Ogbonnaya et al. (2017)
	*wPt-0153*	2D	Ogbonnaya et al. (2017)
	*wPt-733777*	1A	Ogbonnaya et al. (2017)
	*wPt-742925*	5A	Ogbonnaya et al. (2017)
	*wPt-4229*	6A	Ogbonnaya et al. (2017)
Grain weight per spike	*wPt-6709*	1A	Ogbonnaya et al. (2017)
	*wPt-6502*	4A	Ogbonnaya et al. (2017)
Number of grains per spike	*QNGS.cgb-2B*	2B	Wu et al. (2012)
	QNGS.cgb-7A	7A	Wu et al. (2012)
	*S3222159*	2A	Würschum et al. (2018)
	*S1290099*	2A	Würschum et al. (2018)
	*D1280633*	7A	Würschum et al. (2018)
	*D1091178*	4A	Würschum et al. (2018)
	*D1056474*	3B	Würschum et al. (2018)
	*Rht*-*D1*	4D	Würschum et al. (2018)
	*wPt-730427*	2D	Ogbonnaya et al. (2017)
	wPt-731291	7A	Ogbonnaya et al. (2017)
	*qNGS-2B*	2B	Ehdaie et al. (2016)
Spike length	*Xbcd1150–Xbarc61*	1A	Yu et al. (2014)
	*Xmwg912–Xbarc80*	1B	Yu et al. (2014)
	*D3027644*	2A	Würschum et al. (2018)
	*S1006957 (Rht24)*	6A	Würschum et al. (2018)
	*D1128060*	3B	Würschum et al. (2018)
	*D1109894*	6B	Würschum et al. (2018)
	*D3064960*	6B	Würschum et al. (2018)
	*D1109894*	6B	Würschum et al. (2018)
	*Ppd-D1*	2D	Würschum et al. (2018)
	*wPt-2872*	1A	Ogbonnaya et al. (2017)
Spike fertility	*QPFSS.cgb-5A.2*	5A	Wu et al. (2012)
	*QPFSS.cgb-7A*	7A	Wu et al. (2012)
	*Rht-B1*	4B	Würschum et al. (2018)
	*Rht*-*D1*	4D	Würschum et al. (2018)
	*S1019498*	2A	Würschum et al. (2018)
	*D1253047*	4A	Würschum et al. (2018)
Spikelets number per spike	*D1108041*	2A	Würschum et al. (2018)
	*D1128642*	7A	Würschum et al. (2018)
	*D1082846*	7A	Würschum et al. (2018)
	*S2255090*	7A	Würschum et al. (2018)
	*Ppd-D1*	2D	Würschum et al. (2018)
	*D1208470*	5D	Würschum et al. (2018)
	*wPt-732556*	1D	Ogbonnaya et al. (2017)
	*wPt-5787*	5A	Ogbonnaya et al. (2017)
	*wPt-4295*	5D	Ogbonnaya et al. (2017)
	*wPt-5742*	7A	Ogbonnaya et al. (2017)
	*wPt-2883*	7B	Ogbonnaya et al. (2017)
	*wPt-4315*	7D	Ogbonnaya et al. (2017)
	*wPt-731810*	7D, 3B	Ogbonnaya et al. (2017)
Spike compactness	*D1221700*	4A	Würschum et al. (2018)
	*S1089640*	5A	Würschum et al. (2018)
	*D1109152 (Rht24)*	6A	Würschum et al. (2018)
	*D2254379*	7A	Würschum et al. (2018)
	*D1100166*	2D	Würschum et al. (2018)
	S997641	7A	Würschum et al. (2018)
	S2255090	7A	Würschum et al. (2018)
Number of productive tillers	*Qltn.sicau-2B*	2B	Wang et al. (2016)
	*Qltn.sicau-5A*	5A	Wang et al. (2016)
	*QTn.mst-6B*	6B	Naruoka et al. (2011)
	*Qltn.sicau-2D*	2D	Wang et al. (2016)
	*qNTP-4A*	4A	Ehdaie et al. (2016)
	*qNTP-6A*	6A	Ehdaie et al. (2016)
	*qNTP-7B*	7B	Ehdaie et al. (2016)
	*qNTP-3A*	3A	Ehdaie et al. (2016)
Flag leaf length	*QFLL-2B & QFLL-3A*	2B & 3A	Liu et al. (2018a)
	*QFLL*-*4B.1*	4B	Liu et al. (2018a)
	*QFLL*-*4B.2*	4B	Liu et al. (2018a)
	*QFLL*-*5A.1*	5A	Liu et al. (2018a)
	*QFLL*-*5A.2*	5A	Liu et al. (2018a)
	*QFLL*-*5A.3*	5A	Liu et al. (2018a)
	*qFll-1B.1*	*1B*	Fan et al. (2015)
	*qFll-2B.2*	2B	Fan et al. (2015)
	*qFll-1B.2*	1B	Fan et al. (2015)
Flag leaf width	*QFLW-4B.1*	4B	Liu et al. (2018a)
	*QFLW*-*4B.1*	4B	Liu et al. (2018a)
	*QFLW*-*4B.2*	4B	Liu et al. (2018a)
	*qFlw-4B.3*	4B	Fan et al. (2015)
	*qFlw-6B.2*	6B	Fan et al. (2015)
	*qFll-1B.3*	1B	Fan et al. (2015)
	*qFll-2B.1*	2B	Fan et al. (2015)
	*qFll-2B.2*	2B	Fan et al. (2015)
	*qFll-4A.1*	4A	Fan et al. (2015)
	*qFll-4B.1*	4B	Fan et al. (2015)
Flag leaf area	*QFLA-5A.1*	5A	Liu et al. (2018a)
	*QFLA*-*5A.2*	5A	Liu et al. (2018a)
	*QFLA*-*2B*	2B	Liu et al. (2018a)
	*QFLA*-*4B.1*	4B	Liu et al. (2018a)
	*QFLA*-*4B.2*	4B	Liu et al. (2018a)
	*qFla-1B.2*	*1B*	Fan et al. (2015)
	*qFla-2B*	*2B*	Fan et al. (2015)
	*qFla-4B.1*	*4B*	Fan et al. (2015)
	*qFla-5B*	*5B*	Fan et al. (2015)
	*qFla-6B.1*	*6B*	Fan et al. (2015)
	*qFLA-3A*	3A	Ehdaie et al. (2016)
Flag leaf angle	*QFLANG-1B.1*	1B	Liu et al. (2018a)
	*QFLANG-1B.2*	1B	Liu et al. (2018a)
	*QFLANG-3D*	3D	Liu et al. (2018a)
	*QFLANG-4B*	4B	Liu et al. (2018a)
	*QFLANG-6B.1*	6B	Liu et al. (2018a)
	*QFLANG-6B.2*	6B	Liu et al. (2018a)
	*QFLANG-7B*	7B	Liu et al. (2018a)
	*QFLANG-7D*	7D	Liu et al. (2018a)
Grain yield	*wPt-7883*	2B	Ogbonnaya et al. (2017)
	*wPt-664276*	6B	Ogbonnaya et al. (2017)
	*wPt-6832*	1B	Ogbonnaya et al. (2017)
	*qGY-2B*	2B	Ehdaie et al. (2016)
	*QYld.dms-1D1*	1D	Kamran et al. (2013b)
	*QYld.dms-5B1*	5B	Kamran et al. (2013b)
	*QYld.dms-5B2*	5B	Kamran et al. (2013b)
	*IWB72516*	2A	Sukumaran et al. (2018)
	*IWB54923*	3B	Sukumaran et al. (2018)
	*IWA939*	3B	Sukumaran et al. (2018)
	*IWB6308*	4A	Sukumaran et al. (2018)
	*IWA2963*	4B	Sukumaran et al. (2018)
	*IWB52628*	6A	Sukumaran et al. (2018)
	*IWB8615*	7A	Sukumaran et al. (2018)
Root elongation rate	*qER-1*	5D	Hamada et al. (2012)
	*qER-2*	7D	Hamada et al. (2012)
Maximum root length	*qMRL-2B*	2B	Ren et al. (2012)
	*qMRL-7B1*	7B	Ren et al. (2012)
	*QMrl-2A.1*	2A	Kabir et al. (2015)
	*QMrl-2A.2*	2A	Kabir et al. (2015)
	*QMrl-2A.3*	2A	Kabir et al. (2015)
	*QMrl-4D.1*	4D	Kabir et al. (2015)
	*QMrl-4D.2*	4D	Kabir et al. (2015)
	*QMrl-5A.1*	5A	Kabir et al. (2015)
	*QMrl-5A.2*	5A	Kabir et al. (2015)
	*QMrl-6A*	6A	Kabir et al. (2015)
	*QMrl-7B.1*	7B	Kabir et al. (2015)
	*QMrl-7B.2*	7B	Kabir et al. (2015)
	*QMrl-3B.1*	3B	Kabir et al. (2015)
	*QMrl-3B.2*	3B	Kabir et al. (2015)
	*QMrl-5B*	5B	Kabir et al. (2015)
Primary root length	*qPRL-2B1*	2B	Ren et al. (2012)
	*qPRL-2B2*	2B	Ren et al. (2012)
	*qPRL-7B*	7B	Ren et al. (2012)
	*QMrl-2A.1*	2A	Kabir et al. (2015)
	*QMrl-2A.2*	2A	Kabir et al. (2015)
	*QMrl-2A.3*	2A	Kabir et al. (2015)
	*QMrl-4D.1*	4D	Kabir et al. (2015)
Lateral root length	*qLRL-1A*	1A	Ren et al. (2012)
	*qLRL-2B*	2B	Ren et al. (2012)
	*qLRL-4B*	4B	Ren et al. (2012)
	*qLRL-6A*	6A	Ren et al. (2012)
	*qLRL-6B1*	6B	Ren et al. (2012)
	*qLRL-6B2*	6B	Ren et al. (2012)
Total root length	*qTRL-2B*	2B	Ren et al. (2012)
	*qTRL-4B*	4B	Ren et al. (2012)
	*qTRL-6A*	6A	Ren et al. (2012)
	*qTRL-6B*	6B	Ren et al. (2012)
	*qTRL-6D*	6D	Ren et al. (2012)
	*QTrl-2A.1*	2A	Kabir et al. (2015)
	*QTrl-2A.2*	2A	Kabir et al. (2015)
	*QTrl-2B*	2B	Kabir et al. (2015)
	*QTrl-3A.1*	3A	Kabir et al. (2015)
	*QTrl-3A.2*	3A	Kabir et al. (2015)
	*QTrl-3A.3*	3A	Kabir et al. (2015)
	*QTrl-3A.4*	3A	Kabir et al. (2015)
	*QTrl-4D*	3A	Kabir et al. (2015)
	*QTrl-5A.1*	5A	Kabir et al. (2015)
	*QTrl-5A.2*	5A	Kabir et al. (2015)
Total root length	*QTrl-2B*	2B	Kabir et al. (2015)
	*QTrl-3B*	3B	Kabir et al. (2015)
	*QTrl-4A*	4A	Kabir et al. (2015)
	*QTrl-4D*	4D	Kabir et al. (2015)
	*QTrl-6D*	6D	Kabir et al. (2015)
Seminal root angle	*qSRA-6A*	6A	Alahmad et al. (2019)
Root angle, length, number	*Root_MQTL_18*	2A	Soriano and Alvaro (2019)
	*Root_MQTL_2*	1A	Soriano and Alvaro (2019)
	*Root_MQTL_8*	1B	Soriano and Alvaro (2019)
	*Root_MQTL_22*	2B	Soriano and Alvaro (2019)
	*Root_MQTL_25*	2B	Soriano and Alvaro (2019)
	*Root_MQTL_40*	3B	Soriano and Alvaro (2019)
	*Root_MQTL_52*	4B	Soriano and Alvaro (2019)
	*Root_MQTL_69*	6A	Soriano and Alvaro (2019)
Root: shoot ratio	*Root_MQTL_18*	2A	Soriano and Alvaro (2019)
Shallow root weight	*qSRW-3A*	3A	Ehdaie et al. (2016)
	*qSRW-2A*	2A	Ehdaie et al. (2016)
	*qSRW-2D*	2D	Ehdaie et al. (2016)
	*qSRW-4A*	4A	Ehdaie et al. (2016)
Deep root weight	*qDRW-1B*	1B	Ehdaie et al. (2016)
	*qDRW-4B1*	4B	Ehdaie et al. (2016)
	*qDRW-4B2*	4B	Ehdaie et al. (2016)
	*qDRW-2D*	2D	Ehdaie et al. (2016)
	*qDRW-3A*	3A	Ehdaie et al. (2016)
	*qDRW-4A*	4A	Ehdaie et al. (2016)
Root biomass	*qRBio-3A*	3A	Ehdaie et al. (2016)

Maqsood et al. (2017) identified QTL linked to relative water content, cell wall membrane thermo-stability, and photosynthetic rate. Christopher et al. (2018) identified several QTLs associated with the stay-green trait in wheat. QTLs for photosynthetic rate were identified on chromosomes 2A and 7D (llyas et al., 2014). In certain instances, genetic regions linked to physiological traits (e.g. stay-green) were co-located with QTL for yield–related traits yield (Acuna-Galindo et al., 2014). Genomic regions have also been reported for grain yield, thousand kernel weight, biomass, and days to heading which suggested that a group of linked and (or) co-located QTL affected phenological and yield-related traits (Tahmasebi et al., 2016). QTL involved in days to heading and thousand grain weight suggested that early maturity would favour the post-anthesis grain-filling periods and increased grain size and grain yield (Tahmasebi et al., 2016). QTL for chlorophyll content, WUE, photosynthetic rate, and internal CO_2_ concentration were co-located with QTL for grain yield and/or yield components (Xu et al., 2017). QTL which simultaneously control flag leaf traits and yield related traits have also been identified on chromosomes 1B, 2D, 4A, 4D, 4B, 5A, 5B, 6B, 6D, and 7D in wheat (Fan et al., 2015; Wu et al., 2016). Such pleiotropic effects are useful to understand relationships among QTLs and pyramiding favourable alleles in different genetic loci (Hai et al., 2008). Marker-assisted recurrent selection involving pyramiding of important QTL can improve grain yield potential in wheat (Gahlaut et al., 2017). Generally, QTL mapped for physiological traits are limited in wheat, only few identified for chlorophyll content, normalized difference in vegetation index (NDVI), and CT (**Table 4**). Though heritability of physiogical traits is relatively low (Chen et al., 2012; Ogbonnaya et al., 2017), their incorporation in breeding programmes may be useful for cultivar development (Lopes et al., 2012). Therefore, to accelerate breeding aimed at improving grain yield genetic gains in wheat, it is important to dissect genomic regions influencing physiological traits and design associated markers for strategic breeding.

**Table 4:** Quantitative trait loci (QTLs) of some physiological traits in wheat.

Trait	QTL name	Chromosome location of QTL	References
Canopy temperature	*4A-wmc048d*	4A	Lopes et al. (2013)
	*6A-gwm617b*	7D	Lopes et al. (2013)
	*C29P13*	7D	Lopes et al. (2013)
Stay-green	*QSG.qgw-3B.1*	3A	Christopher et al. (2018)
	*QSG.qgw–7B*	7B	Christopher et al. (2018)
	*QSG.qgw-1B*	1B	Christopher et al. (2018)
	*QSG.qgw-2A*	2A	Christopher et al. (2018)
	*qSG.qgw-3B.2*	3B	Christopher et al. (2018)
	*QSG.qgw-4A.1*	4A	Christopher et al. (2018)
	*QSG.qgw-4A.2*	4A	Christopher et al. (2018)
	*QSG.qgw-4B*	4B	Christopher et al. (2018)
	*QSG.qgw-4D*	4D	Christopher et al. (2018)
	*QSG.qgw-5B*	5B	Christopher et al. (2018)
Chlorophyll content	*QChlc.cgb-7A*	7A	Yang et al. (2007a)
	*QChlc.cgb-5A-1*	5A	Yang et al. (2007a)
	*QChlc.cgb-1A*	1A	Yang et al. (2007a)
	*QChlc.cgb-5A-2*	5A	Yang et al. (2007a)
Photosynthetic capacity	*QFv/Fm.cgb-3B-1*	3B	Yang et al. (2007a)
	*QFv/Fm.cgb-3B-2*	3B	Yang et al. (2007a)
	*QFv/Fm.cgb-6A*	6A	Yang et al. (2007a)
	*QFv/Fm.cgb-7D-1*	7D	Yang et al. (2007a)
	*QFv/Fm.cgb-1B*	1B	Yang et al. (2007a)
Water-soluble carbohydrates	*QSwscf.cgb-1A.1*	1A	Yang et al. (2007b)
	*QSwscf.cgb-4B.1*	4B	Yang et al. (2007b)
	*QSwscf.cgb-1D.1*	1D	Yang et al. (2007b)
	*QSwscg.cgb-4A*	4A	Yang et al. (2007b)
	*QSwscm.cgb-1A.1*	1A	Yang et al. (2007b)
	*QSwscf.cgb-2D.1*	2D	Yang et al. (2007b)
	*QSwscf.cgb-2D.2*	2D	Yang et al. (2007b)
	*QSwscf.cgb-7B.1*	7B	Yang et al. (2007b)
	*QSwscf.cgb-7D*	7D	Yang et al. (2007b)
	*QSwscm.cgb-6B.1*	6B	Yang et al. (2007b)
Normalized vegetation index	*QNDVI-A.caas-4AL*	4A	Gao et al. (2015)
	*QNDVI-A.caas-3AL*	3A	Gao et al. (2015)
	*QNDVI-A.caas-1BS*	1B	Gao et al. (2015)
	*QNDVI-A.caas-5BL*	5B	Gao et al. (2015)
	*QNDVI-A.caas-5BS.1*	5B	Gao et al. (2015)
	*QNDVI-A.caas-4BS*	4B	Gao et al. (2015)
	*QNDVI-A.caas-4DS*	4D	Gao et al. (2015)
	*QNDVI-A.caas-5AL*	5A	Gao et al. (2015)
	*QNDVI-A.caas-3AL*	3A	Gao et al. (2015)
	*QNDVI-10.caas-2DS*	2D	Gao et al. (2015)
	*QNDVI-10.caas-5AL*	5A	Gao et al. (2015)
	*QNDVI-10.caas-4BS*	4B	Gao et al. (2015)
	*QNDVI-10.caas-5BL*	5B	Gao et al. (2015)
	*QNDVI-10.caas-6BL*	6B	Gao et al. (2015)
	*QNDVI-10.caas-4DS*	4D	Gao et al. (2015)

In conclusion, genetic improvement can be achieved by either direct selection for primary traits such as grain yield or indirectly through selection of secondary traits related to higher grain yield potential. Breeding high-yielding genotypes incorporating yield-promoting agronomic and physiological traits has accelerated yield gains in wheat. As a result, further grain yield improvement will likely be achieved through in/direct selection targeting yield-related agronomic and physiological attributes. Furthermore, QTL associated with agronomic and physiological traits linked to grain yield are useful for marker-assisted selection of high-performing wheat genotypes.

## Author Contributions

All authors listed have made substantial, direct and intellectual contribution to the work, and approved it for publication.

## Conflict of Interest

The authors declare that the research was conducted in the absence of any commercial or financial relationships that could be construed as a potential conflict of interest.
